# 6-(4-Chloro­phen­yl)-3-methyl­imidazo[2,1-*b*]thia­zole

**DOI:** 10.1107/S1600536813028833

**Published:** 2013-10-26

**Authors:** Alexander S. Bunev, Elena V. Sukhonosova, Vladimir E. Statsyuk, Gennady I. Ostapenko, Victor N. Khrustalev

**Affiliations:** a Department of Chemistry and Chemical Technology, Togliatti State University, 14 Belorusskaya St, Togliatti 445667, Russian Federation; b Department of Organic, Bioorganic and Medicinal Chemistry, Samara State University, 1 Academician Pavlov St, Samara 443011, Russian Federation; c X-Ray Structural Centre, A. N. Nesmeyanov Institute of Organoelement Compounds, Russian Academy of Sciences, 28 Vavilov Street, B-334, Moscow 119991, Russian Federation

## Abstract

In the title compound, C_12_H_9_ClN_2_S, the imidazo[2,1-*b*]thia­zole fragment is planar (r.m.s. deviation = 0.003 Å), and the benzene ring is twisted slightly [by 5.65 (6)°] relative to this moiety. In the crystal, mol­ecules are linked by π–π stacking inter­actions into columns along [010]. The mol­ecules within the columns are arranged alternatively by their planar rotation of 180°. Thus, in the columns, there are the two types of π–π stacking inter­actions, namely, (i) between two imidazo[2,1-*b*]thia­zole fragments [inter­planar distance = 3.351 (2) Å] and (ii) between an imidazo[2,1-*b*]thia­zole fragment and the phenyl ring [inter­planar distance = 3.410 (5) Å]. There are no short contacts between the columns.

## Related literature

For the synthesis and properties of related compounds containing an imidazo[2,1-*b*]thia­zole moiety, see: Raeymaekers *et al.* (1966 ▶); Metaye *et al.* (1992 ▶); Carpenter *et al.* (2003 ▶); Milne *et al.* (2007 ▶); Scribner *et al.* (2008 ▶); Chorell *et al.* (2010 ▶); Guzeldemirci & Kucukbasmaci (2010 ▶); Budriesi *et al.* (2011 ▶); Yousefi *et al.* (2011 ▶).
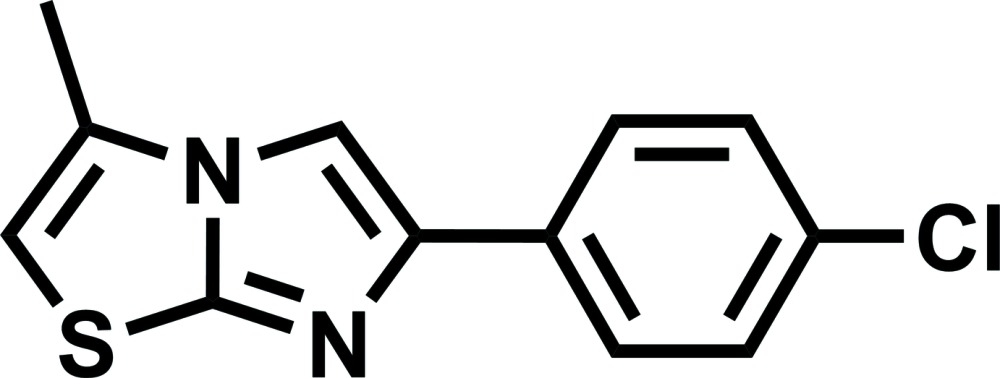


## Experimental

### 

#### Crystal data

C_12_H_9_ClN_2_S
*M* *_r_* = 248.73
Triclinic, 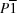

*a* = 7.0624 (5) Å
*b* = 7.7132 (5) Å
*c* = 10.3460 (7) Å
α = 93.353 (1)°
β = 90.107 (1)°
γ = 98.832 (1)°
*V* = 555.92 (7) Å^3^
*Z* = 2
Mo *K*α radiation
μ = 0.50 mm^−1^
*T* = 120 K
0.30 × 0.20 × 0.20 mm

#### Data collection

Bruker APEXII CCD diffractometer
Absorption correction: multi-scan (*SADABS*; Bruker, 2003 ▶) *T*_min_ = 0.864, *T*_max_ = 0.906
6927 measured reflections
2963 independent reflections
2306 reflections with *I* > 2σ(*I*)
*R*_int_ = 0.032

#### Refinement

*R*[*F* ^2^ > 2σ(*F* ^2^)] = 0.038
*wR*(*F* ^2^) = 0.093
*S* = 1.05
2963 reflections
146 parameters
H-atom parameters constrained
Δρ_max_ = 0.38 e Å^−3^
Δρ_min_ = −0.22 e Å^−3^

### 

Data collection: *APEX2* (Bruker, 2005 ▶); cell refinement: *SAINT* (Bruker, 2001 ▶); data reduction: *SAINT*; program(s) used to solve structure: *SHELXTL* (Sheldrick, 2008 ▶); program(s) used to refine structure: *SHELXTL*; molecular graphics: *SHELXTL*; software used to prepare material for publication: *SHELXTL*.

## Supplementary Material

Crystal structure: contains datablock(s) global, I. DOI: 10.1107/S1600536813028833/rk2417sup1.cif

Structure factors: contains datablock(s) I. DOI: 10.1107/S1600536813028833/rk2417Isup2.hkl

Supplementary material file. DOI: 10.1107/S1600536813028833/rk2417Isup3.cml

Additional supplementary materials:  crystallographic information; 3D view; checkCIF report

## Figures and Tables

**Fig. 1.**
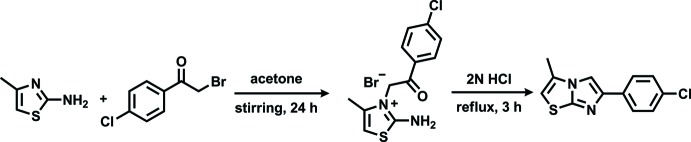
The reaction of 4-methyl-2-aminothiazole with 2-bromo-1-(4-chlorophenyl)ethanone.

**Fig. 2.**
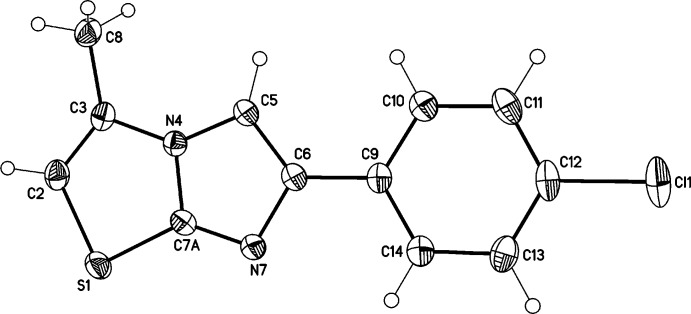
Molecular structure of I with the atom numbering scheme. Displacement ellipsoids are presented at the 50% probability level. H atoms are depicted as small spheres of arbitrary radius.

**Fig. 3.**
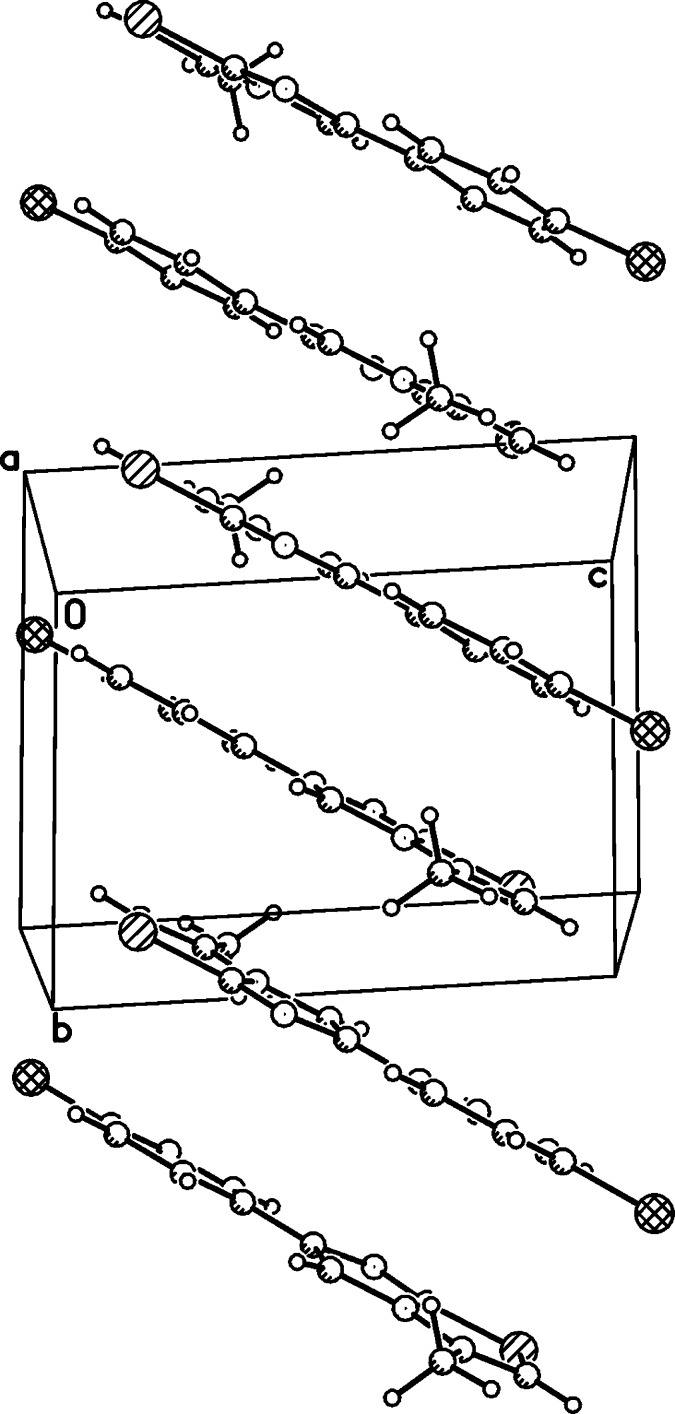
The π···π-bonded column of I along [010].

**Fig. 4.**
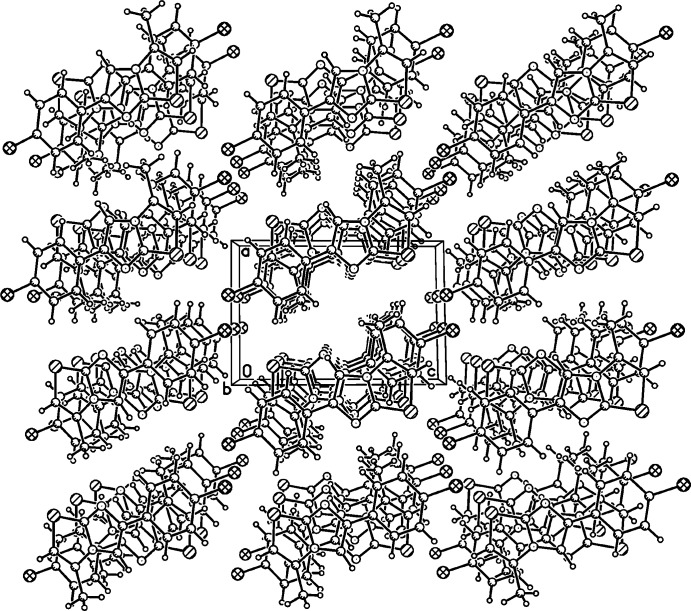
The crystal packing of I along the b axis.

## supplementary crystallographic information

### 1. Comment

Many natural products and biologically active compounds containing imidazo[2,1-*b*]thiazole moieties have been discovered and synthesized so far (Metaye *et al.*, 1992; Guzeldemirci & Kucukbasmaci, 2010; Budriesi *et al.*, 2011). As some typical examples, anthelmintics tetramisole (Raeymaekers *et al.*, 1966), thieno(3,2-*d*)pyrimidinone (Carpenter *et al.*, 2003), 5,6-diarylimidazo[2,1-*b*](1,3)thiazoles (Scribner *et al.*, 2008), C-2 aryl-substituted pilicides (Chorell *et al.*, 2010), and ^11^C-labeled imidazo[2,1-*b*]benzothiazoles (Yousefi *et al.*, 2011) all showed effective biological and medicinal activity. Milne and co-authors (Milne *et al.*, 2007) described the identification and characterization of a molecule bearing imidazo[2,1-*b*]thiazole as an activator of SIRT1, which was structurally unrelated to, and 1000-fold more potent than, resveratrol. And it can bind to the SIRT1 enzyme-peptide substrate complex at an allosteric site amino terminal to the catalytic domain and lower the Michaelis constant for acetylated substrates.

In this work, a 6-(4-chlorophenyl)-3-methylimidazo[2,1-*b*]thiazole, C_12_H_9_ClN_2_S, I, was prepared by the reaction of 4-methyl-2-aminothiazole with 2-bromo-1-(4-chlorophenyl)ethanone (Fig. 1), and its structure was unambiguously established by the X-ray diffraction study (Fig. 2).

The imidazo[2,1-*b*]thiazole fragment in I is planar (r.m.s. deviation is 0.003Å), and the phenyl ring is slightly twisted in relative to this fragment by 5.65 (6)°.

In the crystal, the molecules of I are linked by the intermolecular π···π-stacking interactions into columns along [010] (Fig. 3). The molecules within the columns are arranged alternatively by their planar rotation of 180° (Fig. 3). Thus, in the columns of I, there are the two types of π···π-stacking interactions, namely, (i) between two imidazo[2,1-*b*]thiazole fragments (the interplane distance is 3.351 (2)Å) and (ii) between imidazo[2,1-*b*]thiazole fragment and phenyl ring (the interplane distance is 3.410 (5)Å). There are no any short contacts between the columns (Fig. 4).

### 2. Experimental

A mixture of 4-methyl-2-aminothiazole (5.0 g, 43.8 mmol) and 2-bromo-1-(4-chlorophenyl)ethanone (10.2 g, 43.8 mmol) was dissolved in acetone (40 mL). The reaction mixture was stirred for 24 h. The resulting precipitate was collected, suspended in 2*N* HCl (70 mL) and heated under reflux. The warm solution basified with 20% NH_4_OH yielded the expected imidazo[2,1-*b*]thiazole after cooling upto room temperature. The residue crystallized from *N,N*-dimethylformamide. Yield is 78%. The single crystal of the product was obtained by slow crystallization from *N,N*-dimethylformamide. M.p. = 397-399 K. IR (KBr), ν/cm^-1^: ^1^H NMR (500 MHz, DMSO-*d_6_*, 304 K): 2.43 (s, 3H, CH_3_), 6.91 (s, 1H, H5), 7.46 (d, 2H, *J* = 8.55, H11,13), 7.87 (d, 2H, *J* = 8.55, H10,14), 8.31 (s, 1H, H2) . Anal. Calcd for C_12_H_9_ClN_2_S: C, 57.95; H, 3.65. Found: C, 57.91; H, 3.59.

### 3. Refinement

All hydrogen atoms were placed in the calculated positions with C–H = 0.95Å (for CH-groups) and 0.98Å (for CH_3_-group) and refined in the riding model with fixed isotropic displacement parameters: *U*_iso_(H) = 1.5*U*_eq_(C) for the CH_3_-group and *U*_iso_(H) = 1.2*U*_eq_(C) for the other CH-groups.

### Crystal data

**Table:**

C_12_H_9_ClN_2_S	*Z* = 2
*M**_r_* = 248.73	*F*(000) = 256
Triclinic, *P*1	*D*_x_ = 1.486 Mg m^−^^3^
Hall symbol: -P 1	Melting point = 397–399 K
*a* = 7.0624 (5) Å	Mo *K*α radiation, λ = 0.71073 Å
*b* = 7.7132 (5) Å	Cell parameters from 2226 reflections
*c* = 10.3460 (7) Å	θ = 2.7–30.6°
α = 93.353 (1)°	µ = 0.50 mm^−^^1^
β = 90.107 (1)°	*T* = 120 K
γ = 98.832 (1)°	Prism, colourless
*V* = 555.92 (7) Å^3^	0.30 × 0.20 × 0.20 mm

### Data collection

**Table:**

Bruker APEXII CCD diffractometer	2963 independent reflections
Radiation source: fine-focus sealed tube	2306 reflections with *I* > 2σ(*I*)
Graphite monochromator	*R*_int_ = 0.032
φ and ω scans	θ_max_ = 29.0°, θ_min_ = 2.0°
Absorption correction: multi-scan (*SADABS*; Bruker, 2003)	*h* = −9→9
*T*_min_ = 0.864, *T*_max_ = 0.906	*k* = −10→10
6927 measured reflections	*l* = −14→14

### Refinement

**Table:**

Refinement on *F*^2^	Primary atom site location: structure-invariant direct methods
Least-squares matrix: full	Secondary atom site location: difference Fourier map
*R*[*F*^2^ > 2σ(*F*^2^)] = 0.038	Hydrogen site location: inferred from neighbouring sites
*wR*(*F*^2^) = 0.093	H-atom parameters constrained
*S* = 1.05	*w* = 1/[σ^2^(*F*_o_^2^) + (0.0461*P*)^2^ + 0.0291*P*] where *P* = (*F*_o_^2^ + 2*F*_c_^2^)/3
2963 reflections	(Δ/σ)_max_ = 0.001
146 parameters	Δρ_max_ = 0.38 e Å^−^^3^
0 restraints	Δρ_min_ = −0.22 e Å^−^^3^

### Special details

**Table:**

Geometry. All s.u.'s (except the s.u. in the dihedral angle between two l.s. planes) are estimated using the full covariance matrix. The cell s.u.'s are taken into account individually in the estimation of s.u.'s in distances, angles and torsion angles; correlations between s.u.'s in cell parameters are only used when they are defined by crystal symmetry. An approximate (isotropic) treatment of cell s.u.'s is used for estimating s.u.'s involving l.s. planes.
Refinement. Refinement of *F*^2^ against ALL reflections. The weighted *R*-factor w*R* and goodness of fit *S* are based on *F*^2^, conventional *R*-factors *R* are based on *F*, with *F* set to zero for negative *F*^2^. The threshold expression of *F*^2^ > 2σ(*F*^2^) is used only for calculating *R*-factors(gt) etc. and is not relevant to the choice of reflections for refinement. *R*-factors based on *F*^2^ are statistically about twice as large as those based on *F*, and *R*-factors based on ALL data will be even larger.

### Fractional atomic coordinates and isotropic or equivalent isotropic displacement parameters (Å^2^)

**Table:**

	*x*	*y*	*z*	*U*_iso_*/*U*_eq_	
Cl1	0.38211 (8)	0.73973 (6)	1.00250 (4)	0.04269 (15)	
S1	0.13039 (6)	0.04797 (6)	0.19530 (4)	0.02548 (12)	
C2	−0.1162 (2)	−0.0288 (2)	0.18876 (16)	0.0239 (3)	
H2	−0.1768	−0.1011	0.1184	0.029*	
C3	−0.2120 (2)	0.0238 (2)	0.29218 (15)	0.0203 (3)	
N4	−0.08638 (17)	0.12958 (16)	0.37993 (12)	0.0180 (3)	
C5	−0.0956 (2)	0.21865 (19)	0.49862 (14)	0.0189 (3)	
H5	−0.2063	0.2255	0.5495	0.023*	
C6	0.0896 (2)	0.29557 (19)	0.52765 (14)	0.0190 (3)	
N7	0.21503 (18)	0.25589 (17)	0.42952 (12)	0.0204 (3)	
C7A	0.1018 (2)	0.1576 (2)	0.34408 (15)	0.0201 (3)	
C8	−0.4178 (2)	−0.0182 (2)	0.32472 (17)	0.0258 (3)	
H8A	−0.4875	−0.0863	0.2518	0.039*	
H8B	−0.4704	0.0911	0.3422	0.039*	
H8C	−0.4314	−0.0873	0.4017	0.039*	
C9	0.1606 (2)	0.40574 (19)	0.64315 (14)	0.0188 (3)	
C10	0.0334 (2)	0.4576 (2)	0.73671 (15)	0.0230 (3)	
H10	−0.1006	0.4220	0.7246	0.028*	
C11	0.1012 (3)	0.5600 (2)	0.84631 (16)	0.0272 (4)	
H11	0.0142	0.5955	0.9087	0.033*	
C12	0.2971 (3)	0.6103 (2)	0.86436 (15)	0.0271 (4)	
C13	0.4260 (2)	0.5626 (2)	0.77325 (16)	0.0265 (4)	
H13	0.5598	0.5985	0.7860	0.032*	
C14	0.3567 (2)	0.4617 (2)	0.66341 (15)	0.0222 (3)	
H14	0.4445	0.4298	0.6002	0.027*	

### Atomic displacement parameters (Å^2^)

**Table:**

	*U*^11^	*U*^22^	*U*^33^	*U*^12^	*U*^13^	*U*^23^
Cl1	0.0636 (3)	0.0379 (3)	0.0254 (2)	0.0095 (2)	−0.0140 (2)	−0.01208 (19)
S1	0.0251 (2)	0.0306 (2)	0.0207 (2)	0.00669 (17)	0.00010 (15)	−0.00563 (16)
C2	0.0266 (8)	0.0227 (8)	0.0224 (8)	0.0048 (7)	−0.0059 (6)	−0.0019 (6)
C3	0.0214 (7)	0.0173 (7)	0.0225 (8)	0.0040 (6)	−0.0064 (6)	0.0014 (6)
N4	0.0190 (6)	0.0166 (6)	0.0190 (6)	0.0045 (5)	−0.0015 (5)	0.0008 (5)
C5	0.0220 (7)	0.0181 (7)	0.0172 (7)	0.0053 (6)	0.0011 (6)	0.0017 (6)
C6	0.0230 (8)	0.0173 (7)	0.0176 (7)	0.0054 (6)	0.0003 (6)	0.0019 (6)
N7	0.0199 (6)	0.0221 (6)	0.0191 (6)	0.0034 (5)	0.0004 (5)	−0.0007 (5)
C7A	0.0211 (7)	0.0215 (7)	0.0187 (7)	0.0064 (6)	0.0011 (6)	0.0007 (6)
C8	0.0212 (8)	0.0263 (8)	0.0292 (9)	0.0027 (7)	−0.0041 (6)	−0.0027 (7)
C9	0.0253 (8)	0.0142 (7)	0.0172 (7)	0.0028 (6)	−0.0018 (6)	0.0031 (6)
C10	0.0275 (8)	0.0190 (7)	0.0230 (8)	0.0049 (6)	0.0014 (6)	0.0027 (6)
C11	0.0417 (10)	0.0228 (8)	0.0192 (8)	0.0107 (7)	0.0030 (7)	0.0021 (6)
C12	0.0438 (10)	0.0193 (7)	0.0178 (7)	0.0047 (7)	−0.0072 (7)	−0.0019 (6)
C13	0.0321 (9)	0.0218 (8)	0.0249 (8)	0.0010 (7)	−0.0067 (7)	0.0027 (7)
C14	0.0262 (8)	0.0199 (7)	0.0200 (7)	0.0019 (6)	0.0000 (6)	0.0022 (6)

### Geometric parameters (Å, º)

**Table:**

Cl1—C12	1.7450 (17)	C8—H8A	0.9800
S1—C7A	1.7399 (16)	C8—H8B	0.9800
S1—C2	1.7512 (17)	C8—H8C	0.9800
C2—C3	1.343 (2)	C9—C14	1.397 (2)
C2—H2	0.9500	C9—C10	1.404 (2)
C3—N4	1.4018 (19)	C10—C11	1.384 (2)
C3—C8	1.483 (2)	C10—H10	0.9500
N4—C7A	1.3685 (19)	C11—C12	1.388 (2)
N4—C5	1.3782 (19)	C11—H11	0.9500
C5—C6	1.376 (2)	C12—C13	1.385 (2)
C5—H5	0.9500	C13—C14	1.383 (2)
C6—N7	1.3996 (18)	C13—H13	0.9500
C6—C9	1.466 (2)	C14—H14	0.9500
N7—C7A	1.315 (2)		
			
C7A—S1—C2	89.68 (7)	H8A—C8—H8B	109.5
C3—C2—S1	113.96 (12)	C3—C8—H8C	109.5
C3—C2—H2	123.0	H8A—C8—H8C	109.5
S1—C2—H2	123.0	H8B—C8—H8C	109.5
C2—C3—N4	110.46 (14)	C14—C9—C10	118.13 (14)
C2—C3—C8	130.15 (15)	C14—C9—C6	120.96 (14)
N4—C3—C8	119.35 (13)	C10—C9—C6	120.91 (14)
C7A—N4—C5	106.48 (12)	C11—C10—C9	120.71 (16)
C7A—N4—C3	115.56 (13)	C11—C10—H10	119.6
C5—N4—C3	137.96 (13)	C9—C10—H10	119.6
C6—C5—N4	105.33 (12)	C10—C11—C12	119.54 (15)
C6—C5—H5	127.3	C10—C11—H11	120.2
N4—C5—H5	127.3	C12—C11—H11	120.2
C5—C6—N7	111.17 (13)	C13—C12—C11	121.06 (15)
C5—C6—C9	128.03 (14)	C13—C12—Cl1	119.50 (14)
N7—C6—C9	120.79 (14)	C11—C12—Cl1	119.43 (13)
C7A—N7—C6	103.37 (12)	C14—C13—C12	118.94 (16)
N7—C7A—N4	113.65 (13)	C14—C13—H13	120.5
N7—C7A—S1	136.01 (12)	C12—C13—H13	120.5
N4—C7A—S1	110.33 (11)	C13—C14—C9	121.60 (15)
C3—C8—H8A	109.5	C13—C14—H14	119.2
C3—C8—H8B	109.5	C9—C14—H14	119.2
			
C7A—S1—C2—C3	−0.51 (13)	C3—N4—C7A—S1	−0.54 (16)
S1—C2—C3—N4	0.30 (17)	C2—S1—C7A—N7	179.01 (17)
S1—C2—C3—C8	−177.40 (14)	C2—S1—C7A—N4	0.57 (12)
C2—C3—N4—C7A	0.17 (19)	C5—C6—C9—C14	174.39 (15)
C8—C3—N4—C7A	178.15 (13)	N7—C6—C9—C14	−5.4 (2)
C2—C3—N4—C5	−179.57 (16)	C5—C6—C9—C10	−5.4 (2)
C8—C3—N4—C5	−1.6 (3)	N7—C6—C9—C10	174.78 (14)
C7A—N4—C5—C6	−0.14 (16)	C14—C9—C10—C11	−0.6 (2)
C3—N4—C5—C6	179.61 (16)	C6—C9—C10—C11	179.28 (14)
N4—C5—C6—N7	−0.20 (17)	C9—C10—C11—C12	−0.7 (2)
N4—C5—C6—C9	−179.99 (14)	C10—C11—C12—C13	1.2 (2)
C5—C6—N7—C7A	0.46 (16)	C10—C11—C12—Cl1	179.89 (12)
C9—C6—N7—C7A	−179.73 (13)	C11—C12—C13—C14	−0.6 (2)
C6—N7—C7A—N4	−0.56 (17)	Cl1—C12—C13—C14	−179.23 (12)
C6—N7—C7A—S1	−178.95 (14)	C12—C13—C14—C9	−0.7 (2)
C5—N4—C7A—N7	0.46 (17)	C10—C9—C14—C13	1.2 (2)
C3—N4—C7A—N7	−179.35 (12)	C6—C9—C14—C13	−178.60 (14)
C5—N4—C7A—S1	179.27 (10)		
